# Association between a stepwise health education model based on evidence-based practice and self-care behaviors of patients with chronic heart failure: a single-center retrospective cohort study

**DOI:** 10.3389/fcvm.2026.1932900

**Published:** 2026-10-14

**Authors:** Yu Chang, Lili Wang, Xiaoqian Hao, Yajing Hu, Fujuan Sun

**Affiliations:** 1 Department of Cardiology, Daxing District Hospital of Traditional Chinese Medicine and Western Medicine, Beijing, China; 2 Department of Emergency Medicine, Daxing District Hospital of Traditional Chinese Medicine and Western Medicine, Beijing, China; 3 Operating Room, Daxing District Hospital of Traditional Chinese Medicine and Western Medicine, Beijing, China

**Keywords:** cardiac function, chronic heart failure, evidence-based practice, readmission rate, self-care, stepwise health education

## Abstract

**Objective:**

This study aims to investigate the association between a stepwise health education model based on evidence-based practice (EBP) and self-care behaviors, cardiac function improvement, and clinical outcomes in patients with chronic heart failure (CHF).

**Methods:**

A retrospective analysis was performed on the clinical data of 120 patients with CHF admitted to our hospital from January 2023 to December 2024. The patients were divided into a control group (CG, *n* = 62, receiving routine health education) and an intervention group (IG, *n* = 58, receiving stepwise health education based on EBP in addition to routine care) according to the health education strategy received. The 6-month total score of the EHFScB-9 was defined as the primary outcome. Cardiac function indicators (LVEF, BNP, and 6MWD), biochemical indicators, clinical outcomes, individual scale items, and hospitalization-related indicators were considered exploratory outcomes. Factors associated with the occurrence of composite endpoint events were analyzed.

**Results:**

After 6 months of intervention, the IG exhibited significantly higher LVEF and 6MWD and significantly lower BNP level than the CG (*P* < 0.05). The serum sodium level in the IG was significantly higher than that in the CG (*P* < 0.05). Exploratory analyses showed that the nominal *P* values for the 30-day, 90-day, and 6-month readmission rates, as well as the incidence of composite endpoint events, were all <0.05 in the IG. The average length of stay and total hospitalization costs in the IG were significantly lower than those in the CG (*P* < 0.05); however, these findings were interpreted as exploratory. At the 6-month follow-up, the IG exhibited lower total EHFScB-9 scores and lower scores for individual items than the CG (*P* < 0.05), indicating better self-care behaviors. Comparisons of individual items were exploratory findings. Multivariable analysis revealed that NYHA class III-IV (OR = 3.765), concomitant atrial fibrillation (OR = 2.440), LVEF < 40% (OR = 3.184), and lack of stepwise health education based on EBP (OR = 2.607) were associated with the composite endpoints.

**Conclusion:**

In this single-center retrospective cohort study, analyses without adjustment for multiple comparisons suggested that a step health education model based on evidence-based practice was associated with better self-care behaviors, improved cardiac function, and lower rates of readmission and composite endpoint events.

## 1 Introduction

Chronic heart failure (CHF) is the end stage of various cardiac diseases and is characterized by decreased cardiac pump function and inadequate cardiac output to meet the body's metabolic demands. It is one of the main causes of death in patients with cardiovascular diseases (1, 2). Epidemiological data indicate that the prevalence of heart failure in China is approximately 0.9%, effecting more than 12 million patients (3). In addition, with the accelerated aging of the population, the incidence of CHF shows a rising trend year by year (3). Despite significant progress in drug and device therapy in recent years, the prognosis of CHF patients remains poor, with a 5-year mortality rate exceeding 50% and a persistently high rate of repeated hospitalizations, which seriously affects the quality of life of patients and imposes a heavy socioeconomic burden (4, 5).

Self-care behavior is a key factor affecting the prognosis of CHF patients. Research (6) indicates that good self-care behavior helps patients identify early signs of worsening symptoms and promptly adopt appropriate measures, including seeking medical attention, making lifestyle modifications, and adhering to prescribed medications, thereby reducing the occurrence of acute decompensation events. However, conventional health education is often delivered using relatively standardized content and formats and may not adequately incorporate patients’ health literacy, cognitive ability, disease status, family support, and individual preferences when determining educational priorities, frequency, and delivery methods. As a result, patients’ disease knowledge, medication adherence, and lifestyle management may still require improvement (7). Evidence-based practice (EBP) emphasizes the integration of the best available evidence, clinical expertise and resources of healthcare professionals, available organizational resources, and patients’ and families’ values, preferences, and circumstances to inform nursing decision-making (8). Applying EBP to health education for patients with CHF may help enable the scientific rigor, clinical feasibility, and alignment with individual patient needs of educational content. Stepwise health education, which is delivered progressively according to patients’ cognitive levels, learning capacity, and disease stages, may improve learning outcomes and knowledge retention (9). In recent years, studies have explored the application of EBP and stepwise health education in CHF management (10); however, evidence regarding the development of educational interventions, individualized tailoring, and long-term outcomes remains limited.

Therefore, this study conducted a retrospective cohort analysis to investigate the association between a stepwise health education model based on EBP and self-care behaviors, cardiac function, and clinical outcomes in patients with CHF, and to explore factors associated with composite endpoint events, with the aim of providing evidence for optimizing health education strategies in patients with CHF.

## 2 Materials and methods

### 2.1 Study design

This was a single-center retrospective cohort study reviewed and approved by the ethics committee of Daxing District Hospital of Traditional Chinese Medicine and Western Medicine. The study protocol adhered to the ethical requirements of the Declaration of Helsinki. Given the retrospective nature of the study, the ethics committee agreed to waive patient informed consent.

### 2.2 Study population

Patients with CHF who were admitted to our institution between January 2023 and December 2024 were retrospectively identified using the hospital information system according to predefined inclusion and exclusion criteria.

Inclusion criteria: (1) age ≥ 18 years; (2) in accordance with the diagnostic criteria of the Chinese Guidelines for the Diagnosis and Treatment of Heart Failure 2018 (11); (3) New York Heart Association (NYHA) (12) functional class II-IV; (4) disease duration ≥ 6 months; (5) normal cognitive function and ability to cooperate with health education; (6) complete medical records, including cardiac function and laboratory test results at baseline and during follow-up; (7) follow-up time ≥ 6 months.

Exclusion criteria: (1) acute cardiac events such as acute myocardial infarction and acute myocarditis; (2) concurrent malignant tumors or severe hepatic and renal insufficiency; (3) concurrent severe cognitive impairment or psychiatric disorders; (4) patients with expected survival less than 6 months; (5) pregnant or lactating women; (6) loss to follow-up or incomplete data during follow-up; (7) concurrent participation in other clinical studies.

Sample size consideration: This was a retrospective cohort study with a predefined study period. All eligible patients with complete clinical data between January 2023 and December 2024 were included. Therefore, no prospective sample size calculation was performed, and the final sample size was determined by the number of available eligible cases within the study period. Sensitivity analysis indicated that, with a two-sided *α* level of 0.05 and sample sizes of 62 patients in the control group (CG) and 58 patients in the intervention group (IG), the available sample size provided 80% power to detect an effect size of approximately 0.52 standard deviations in comparisons between two independent groups. This calculation was performed only to describe the detectable effect range and was not intended to replace a prospective sample size calculation.

### 2.3 Grouping method

This was a retrospective study with non-randomized exposure groups, and no random allocation was performed by the investigators. Patients were classified according to the health education strategies they actually received, based on information documented in medical records and nursing records. Patients who received routine health education were categorized into the CG, whereas those who received a stepwise health education model based on EBP in addition to routine care were categorized into the IG. The health education strategies were delivered by the assigned nursing teams during hospitalization according to the routine clinical pathways at that time. Because the medical records did not allow complete reconstruction of all factors influencing the selection of different education strategies for each patient, residual selection bias cannot be excluded.

Patients in the CG (*n* = 62) received routine health education, including basic disease-related knowledge, medication guidance, dietary advice, and instructions regarding physical activity and rest. Standard discharge education was provided before discharge, followed by routine telephone follow-up in accordance with departmental protocols. Other aspects of care, including vital sign monitoring, symptom assessment, medication administration as prescribed, and basic nursing care, were implemented according to the same clinical pathway.

Patients in the IG (*n* = 58) received stepwise health education based on EBP in addition to the above-mentioned routine care. To enhance reproducibility, this study used the Iowa Model revised of EBP as a framework to provide a structured report on the process of developing and implementing the existing program (13): identifying clinical issues in CHF self-care; forming a multidisciplinary team comprising cardiologists, specialty nurses, dietitians, and physical therapists; searching for and evaluating evidence; integrating the evidence with the hospital's resources, the team's experience, and the preferences and circumstances of patients and their families; developing core educational content; and updating it based on feedback. The educational content was primarily based on CHF guidelines and self-care recommendations (11, 14, 15), covering symptom recognition and identification of warning signs, daily body weight and edema monitoring, medication purposes and adherence, sodium restriction and individualized fluid management, activity and rest recommendations, action plans for symptom worsening, and follow-up arrangements. (1) Adaptation of the stepwise approach to short hospital stays: The progression of educational stages was determined by educational content and patients’ mastery of knowledge and skills rather than by a predefined length of hospitalization. During the first 1–3 days after admission, patients underwent needs assessment and received education on basic heart failure knowledge, warning signs, and body weight monitoring. For patients with stable conditions and longer hospital stays, additional education on dietary management, medication adherence, physical activity, and emergency management was continued during hospitalization. For patients hospitalized for ≤3 days, essential safety information and a written action plan were completed before discharge, while remaining skill-based education was transferred to post-discharge telephone or WeChat-based follow-up and reinforced during the first follow-up visit. After discharge, educational materials were regularly provided, and knowledge and self-care skills were continuously reinforced through follow-up. (2) Individualized implementation: Essential safety information was standardized for all patients, while the healthcare team tailored the educational priorities, delivery pace, repetition frequency, educational media (including one-to-one education, videos, illustrated manuals, or peer education), level of family involvement, and frequency of post-discharge reinforcement according to patients’ educational background and health literacy, cognitive and learning abilities, NYHA class and current symptoms, family support, and accessibility to communication and follow-up. After each educational session, patients’ understanding was assessed through questioning, demonstration of body weight and edema monitoring, and verification of symptom-based action plans. Patients who failed to achieve adequate mastery received repeated education or alternative, more intuitive educational materials. For patients with changes in clinical status, the focus of dietary management, physical activity, and symptom monitoring was adjusted accordingly. (3) Development and refinement of the intervention protocol: A multidisciplinary team developed standardized core educational content based on retrieved clinical guidelines, systematic reviews, and self-care recommendations. The protocol was refined during case follow-up and team discussions by incorporating patient feedback, implementation difficulties, and emerging evidence. In this model, “standardization” referred to mandatory essential safety information and documentation requirements, whereas “individualization” referred to the adjustable aspects of educational priorities, delivery formats, pace, and reinforcement frequency. Since this was a retrospective study, the educational protocol was not independently validated using formal reliability and validity assessments before study initiation.

### 2.4 Outcome measures

#### 2.4.1 Primary outcome measure

The primary outcome was the total score of the European Heart Failure Self-care Behavior Scale-9 (EHFScB-9) (16) at the 6-month follow-up. The EHFScB-9 was assessed at baseline and at 6 months, consisting of 9 items scored from 1 to 5, with a total score ranging from 9 to 45. Lower scores indicated better self-care behaviors. The primary analysis was performed using analysis of covariance (ANCOVA), with the 6-month EHFScB-9 total score as the dependent variable and the baseline total score as a covariate. Adjusted between-group differences, 95% confidence intervals (CIs), and *P* values were reported.

#### 2.4.2 Secondary outcome measures

(1) Cardiac function indicators: Left ventricular ejection fraction (LVEF), B-type natriuretic peptide (BNP), and 6-minute walk distance (6MWD) were assessed at baseline and at the 6-month follow-up. (2) Clinical outcomes: Clinical outcomes included 30-day, 90-day, and 6-month readmission rates, hospitalization for worsening heart failure, emergency department visits, 6-month mortality, and composite endpoint events (defined as readmission, death, or emergency department visits). (3) Biochemical indicators: Serum sodium, serum potassium, creatinine, and hemoglobin levels were measured at baseline and at the 6-month follow-up. (4) Medication use: The use of angiotensin-converting enzyme inhibitors/angiotensin receptor blockers (ACEIs/ARBs), *β*-blockers, diuretics, digitalis, and spironolactone was recorded. Comparisons of individual EHFScB-9 items, cardiac function indicators, biochemical indicators, clinical outcomes, and medication use were considered exploratory analyses.

#### 2.4.3 Exploratory outcomes

Exploratory outcomes included length of hospital stay, total hospitalization costs, ICU admission rate, and incidence of complications during hospitalization. These indicators referred to the hospitalization at cohort inclusion rather than subsequent readmissions. Since exposure to the health education strategy started during this hospitalization, and early ICU admission and part of the length of stay could have occurred before adequate exposure to the educational strategy, these outcomes were analyzed only as exploratory comparisons and were not used for causal inference.

### 2.5 Data collection and quality control

Data were collected independently by two trained researchers using standardized data collection forms. Data were entered and cross-checked by two researchers. Disputed data were adjudicated by a third-party researcher. Cardiac function parameters were assessed by the same team of sonographers, and laboratory parameters were assessed uniformly by the hospital's clinical laboratory to ensure data accuracy and consistency.

### 2.6 Statistical methods

SPSS 26.0 software was used for statistical analysis. Continuous data were tested to be normally distributed after the Shapiro–Wilk test and expressed as mean ± standard deviation (x¯ ± s). The primary outcome was analyzed using ANCOVA, with the 6-month EHFScB-9 total score as the dependent variable, group as the fixed factor, and baseline EHFScB-9 total score as the covariate. Intergroup comparisons of continuous variables at baseline and 6 months were performed using independent-samples t tests and were considered exploratory analyses. Categorical data were expressed as number of cases (percentages). Pearson's chi-square test was used when all expected frequencies were ≥5, whereas a two-sided Fisher's exact test was applied when any expected frequency was <5. Univariable and multivariable logistic regression analyses were performed with composite endpoint events as the dependent variable, and non-receipt of the stepwise health education model based on EBP (i.e., the routine health education group) was included as one of the exposure variables. Only the EHFScB-9 total score was prespecified as the primary outcome. All other comparisons and item-level analyses were considered exploratory, with *P* values reported as nominal *P* values without adjustment for multiplicity. These findings were not used for confirmatory or causal inference. A two-sided *P* value <0.05 was considered statistically significant.

## 3 Results

### 3.1 Comparison of baseline data between the two groups

Baseline clinical data, including sex, age, BMI, disease duration, NYHA classification, and comorbidities, were collected and compared between the two groups. The results showed no statistically significant differences between the two groups in these data (*P* > 0.05), indicating good comparability between the two groups (Table 1).

**Table 1:** Comparison of baseline data between the two groups (x¯ ± s)/ [n (%)].

General data	Control group (*n* = 62)	Intervention group (*n* = 58)	*t/χ^2^*	*P*
Age (years)	68.45 ± 11.32	67.82 ± 10.86	0.315	0.754
Sex (male/female)	35/27	32/26	0.024	0.877
BMI (kg/m^2^)	24.76 ± 3.42	25.13 ± 3.58	0.586	0.559
Disease duration (years)	4.23 ± 2.15	4.46 ± 2.28	0.577	0.565
NYHA classification (II/III/IV)	38/20/4	34/19/5	0.312	0.856
Hypertension [*n* (%)]	47 (75.81)	42 (72.41)	0.179	0.672
Diabetes mellitus [*n* (%)]	28 (45.16)	27 (46.55)	0.023	0.879
Atrial fibrillation [*n* (%)]	22 (35.48)	19 (32.76)	0.102	0.749

### 3.2 Comparison of cardiac function indicators between the two groups

There were no statistically significant differences in LVEF, BNP, and 6MWD between the two groups at baseline (*P* > 0.05). At 6 months after intervention, the IG showed significantly higher LVEF and 6MWD, and significantly lower BNP levels than the CG (*P* < 0.05) (Figure 1). The above comparisons were exploratory analyses without adjustment for multiplicity, and the reported *P* values were nominal *P* values.

**Figure 1:**
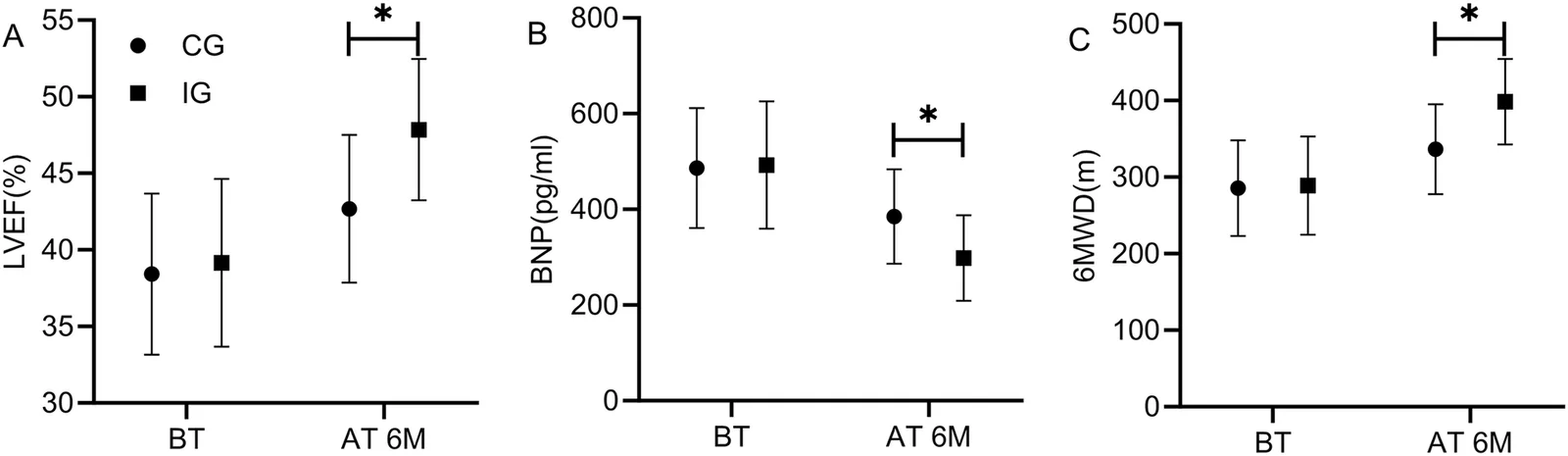
Comparison of cardiac function indicators between the two groups. Before intervention, there were no statistically significant differences in LVEF (figure A), BNP (figure B), and 6MWD (figure C) between the two groups (*P* > 0.05). After 6 months, the IG showed significantly higher LVEF (figure A) and 6MWD (figure C), and significantly lower BNP (figure B) levels compared to the CG (*P* < 0.05). BT, before treatment; AT 6M, at 6 months after treatment. *indicates statistically significant intergroup differences.

### 3.3 Comparison of biochemical indicators between the two groups

Before and after intervention, serum sodium, serum potassium, creatinine, and hemoglobin levels were collected and compared between the two groups. There were no statistically significant differences in these biochemical indicators between the two groups before intervention (*P* > 0.05). At 6 months of intervention, serum sodium levels in the IG were significantly higher than those in the CG (*P* < 0.05). There were no statistically significant differences in other biochemical indicators, such as serum potassium, creatinine, and hemoglobin levels (*P* > 0.05) (Figure 2). The above comparisons were exploratory analyses without adjustment for multiplicity, and the reported *P* values were nominal *P* values.

**Figure 2:**
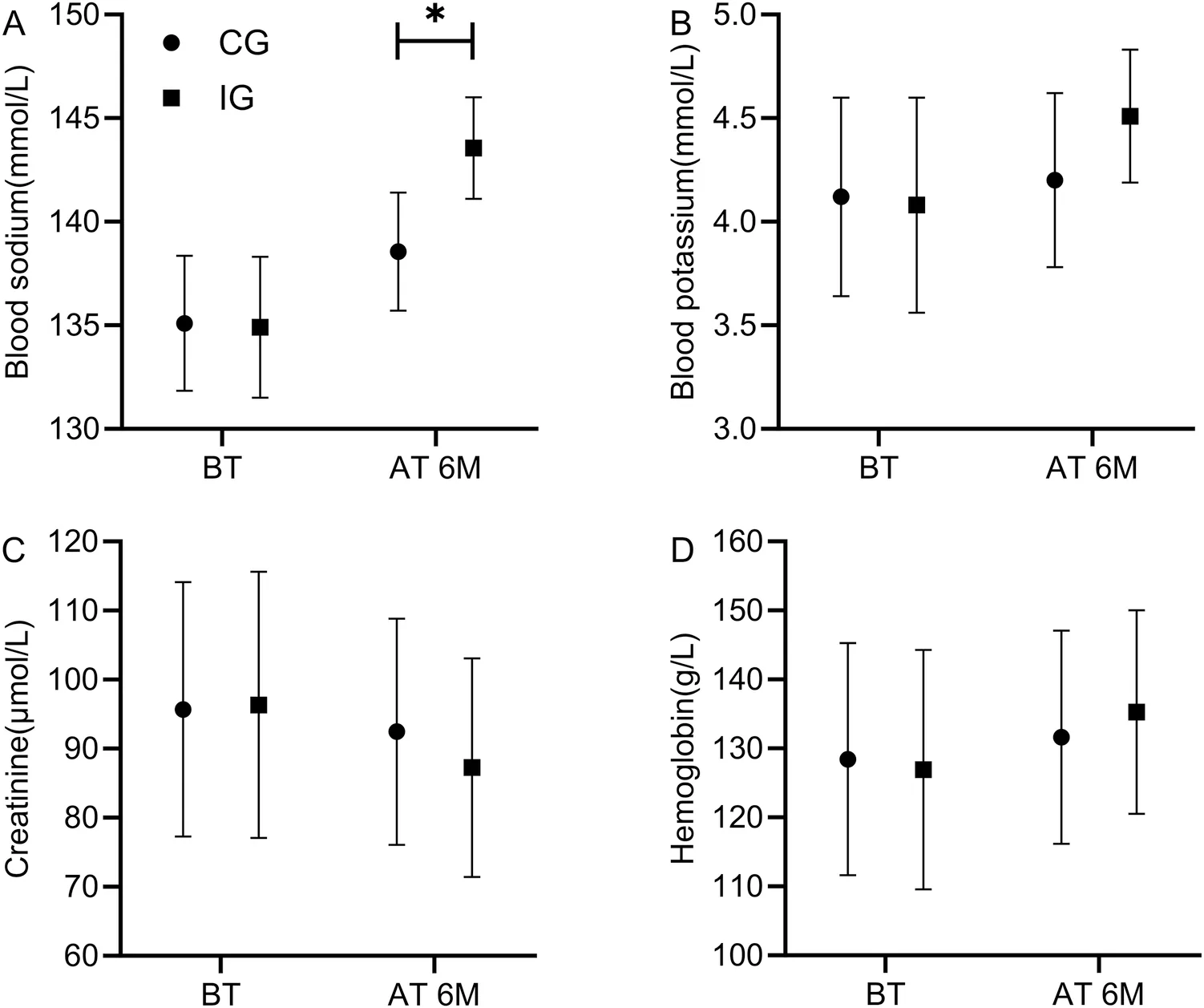
Comparison of biochemical indicators between the two groups. Before intervention, there were no statistically significant differences in serum sodium (figure A), serum potassium (figure B), creatinine (figure C), and hemoglobin (figure D) levels between the two groups (*P* > 0.05). At 6 months, the IG exhibited higher serum sodium levels (figure A) than the CG (*P* < 0.05). BT, before treatment; AT 6M, at 6 months after treatment. *indicates statistically significant intergroup differences.

### 3.4 Comparison of clinical outcomes between the two groups

During the 6-month follow-up, exploratory analyses indicated that the incidences of 30-day, 90-day, and 6-month readmission, hospitalization for worsening heart failure, emergency department visits, and composite endpoint events were lower in the IG than in the CG, with all nominal *P* values <0.05 without adjustment for multiple comparisons. No statistically significant difference was observed in 6-month mortality between the two groups (Table 2). The 30-day readmission rate was compared using Pearson's chi-square test (*χ*^2^ = 4.594, *P* = 0.032). The minimum expected frequency was 6.77. As a sensitivity analysis, a two-sided Fisher's exact test was conducted, yielding a *P* value of 0.045, and the results were consistent.

**Table 2:** Comparison of clinical outcomes between the two groups [*n* (%)].

Clinical outcomes	Control group (*n* = 62)	Intervention group (*n* = 58)	*χ^2^*	*P*
30-day readmission	11 (17.74)	3 (5.17)	4.592	0.032
90-day readmission	18 (29.03)	7 (12.07)	5.228	0.022
6-month readmission	24 (38.71)	10 (17.24)	6.802	0.009
Hospitalization for worsening heart failure	16 (25.81)	6 (10.34)	4.785	0.029
Emergency department visit	28 (45.16)	13 (22.41)	6.894	0.009
6-month mortality	3 (4.84)	1 (1.72)	Fisher	0.619
Composite endpoint events	31 (50.00)	16 (27.59)	6.318	0.012

Composite endpoint events include readmission, death, or emergency department visit. Except for 6-month mortality analyzed using a two-sided Fisher's exact test, all other outcomes were assessed using Pearson's chi-square test. All *P* values are nominal and unadjusted for multiple comparisons; therefore, the findings should be interpreted as exploratory.

### 3.5 Comparison of exploratory outcomes during hospitalization between the two groups

The average length of stay and total hospitalization costs in the IG were significantly lower than those in the CG (*P* < 0.05). While there was a trend toward a decrease in ICU admission rate and the incidence of hospitalization complications, the differences were not statistically significant (*P* > 0.05) (Figure 3). As the health education was initiated during this hospitalization, these observations were considered exploratory and cannot be solely attributed to the educational intervention.

**Figure 3:**
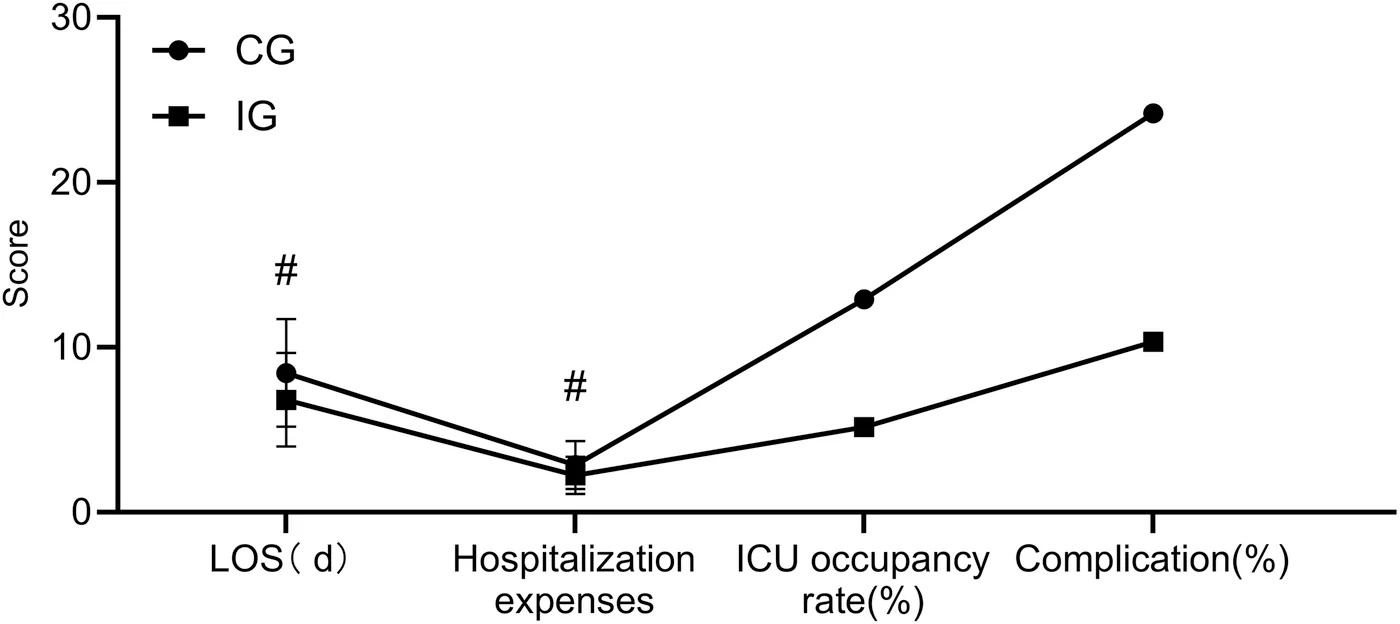
Comparison of hospitalization-related indicators between the two groups. The IG had lower length of stay and hospitalization costs than the CG (*P* < 0.05). There were no statistically significant differences in ICU admission rate and incidence of hospitalization complications between the two groups (*P* > 0.05). LOS, length of stay. *indicates statistically significant intergroup differences.

### 3.6 Comparison of medication use between the two groups

There was no statistically significant difference in the use rates of various medications between the two groups at baseline (*P* > 0.05). At the 6-month follow-up, the use rate of *β*-blockers was significantly higher in the IG than in the CG (*P* < 0.05) (Table 3). The above comparisons were exploratory analyses without adjustment for multiplicity, and the reported *P* values were nominal *P* values.

**Table 3:** Comparison of medication use between the two groups [n (%)].

Medications	Baseline		At the 6-month follow-up		*χ^2^*	*P*
	Control group	Intervention group	Control group	Intervention group		
ACEI/ARB	52 (83.87)	48 (82.76)	54 (87.10)	55 (94.83)	2.141	0.143
*β*-blockers	48 (77.42)	46 (79.31)	50 (80.65)	54 (93.10)*	4.123	0.042
Diuretics	58 (93.55)	54 (93.10)	59 (95.16)	56 (96.55)	0.128	0.721
Digitalis	16 (25.81)	14 (24.14)	15 (24.19)	11 (18.97)	0.459	0.498
Spironolactone	42 (67.74)	38 (65.52)	45 (72.58)	48 (82.76)	1.765	0.184

Compared with the control group, *P < 0.05.

### 3.7 Comparison of self-care scores before and after intervention in the two groups

The EHFScB-9 was used to assess the self-care behavior of the two groups, with lower scores indicating better self-care behaviors. There was no statistically significant difference in baseline scores between the two groups (33.06 ± 5.67 vs. 33.25 ± 4.98, *P* > 0.05). At 6 months, the total score was lower in the IG than in the CG (19.97 ± 3.94 vs. 30.03 ± 4.86, *P* > 0.05). The unadjusted between-group mean difference (IG−CG) was −10.06 points (95% CI: −11.65 to −8.47). Comparisons of individual items were considered exploratory and were not adjusted for multiple comparisons (Figure 4).

**Figure 4:**
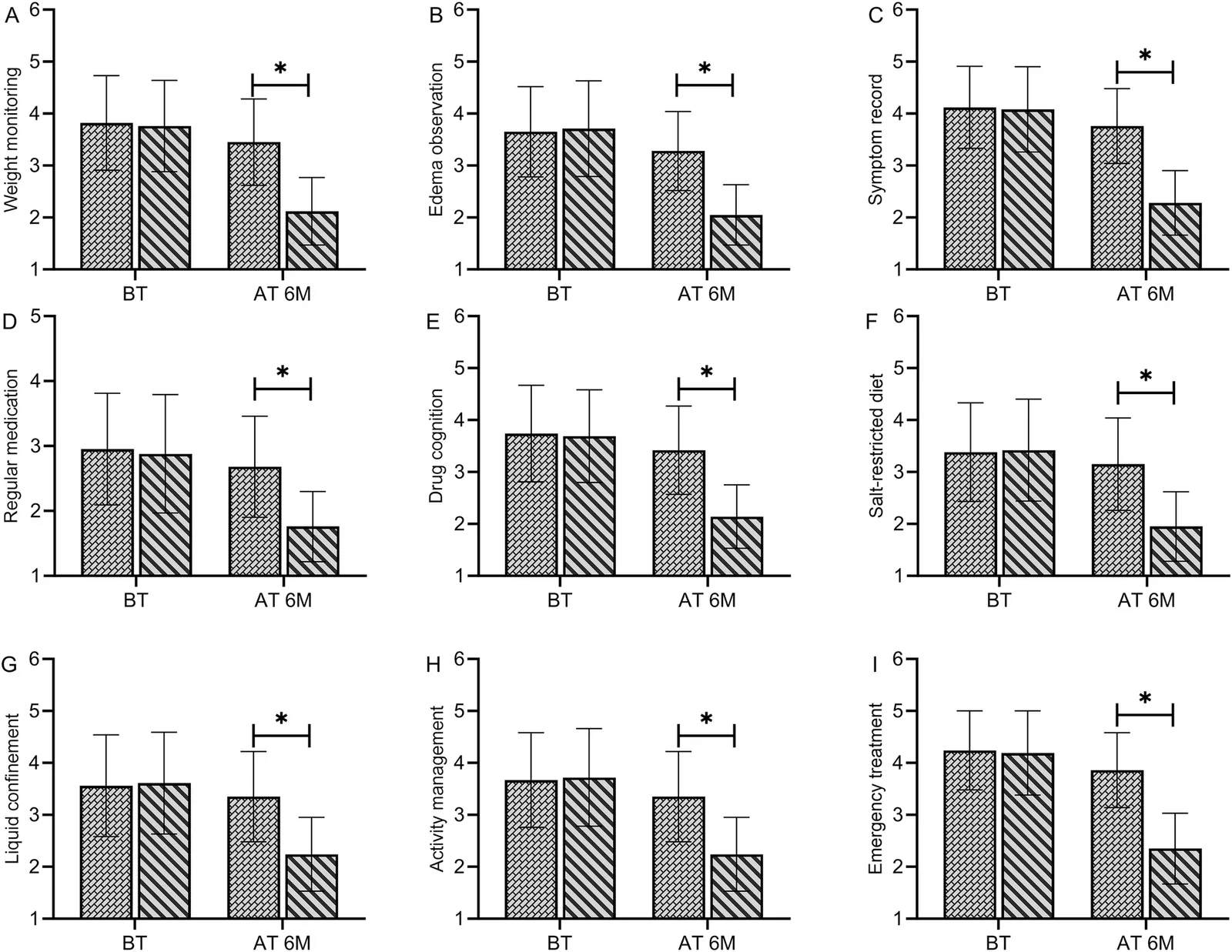
Comparison of individual EHFScB-9 item scores between the two groups at baseline and 6 months. No statistically significant differences were observed in individual item scores between the groups at baseline. At 6 months, scores for weight monitoring (figure A), edema assessment (figure B), symptom recording (figure C), regular medication (figure D), medication awareness (figure E), salt restriction (figure F), fluid restriction (figure G), activity management (figure I), and emergency treatment (figure J) were all lower in the IG than in the CG. *P* values for individual items were not adjusted for multiple comparisons and should be interpreted as exploratory findings. IG, intervention group; CG, control group. *indicates statistically significant intergroup differences. Scoring was performed on a 5-point scale (1 = completely, 2 = often, 3 = sometimes, 4 = rarely, 5 = never), with lower scores indicating better self-care behaviors.

### 3.8 Analysis of factors associated with composite endpoint events

The occurrence of composite endpoint events (readmission, death, or emergency department visit) was used as the dependent variable. Baseline characteristics, including sex, age, BMI, disease duration, comorbidities, and laboratory parameters, were used as independent variables. The risk factors for the composite endpoint events were analyzed. The results of univariable analysis showed that age ≥ 70 years, low education level, disease duration ≥ 5 years, NYHA class III-IV, concomitant diabetes, concomitant atrial fibrillation, LVEF < 40%, BNP ≥ 400 pg/mL, creatinine ≥ 100 μmol/L, blood sodium < 135 mmol/L, hemoglobin < 120 g/L, non-receipt of the stepwise health education model based on EBP (i.e., routine health education group), and no use of *β*-blockers were associated with the occurrence of composite endpoint events (*P* < 0.1) (Table 4). Further multivariable logistic regression analysis based on the above factors revealed that NYHA class III-IV, concomitant atrial fibrillation, LVEF < 40%, and non-receipt of the stepwise health education model based on EBP (i.e., routine health education group) were identified as independent factors associated with composite endpoint events (*P* < 0.05) (Table 5). The dichotomization thresholds for continuous variables were selected to enhance clinical interpretability. BNP ≥400 pg/mL was used as a clinically relevant threshold for elevated BNP levels, while creatinine ≥100 μmol/L was defined according to the upper limit of the adult reference range provided by the hospital laboratory. Neither threshold was determined through data-driven selection based on study outcomes. Given that dichotomization of continuous variables may result in information loss, these analyses were considered exploratory and should be interpreted cautiously.

**Table 4:** Univariable analysis of factors associated with composite endpoint events.

Variables	Occurrence group (*n* = 47)	Non-occurrence group (*n* = 73)	*t/χ^2^*	*P*
Demographic characteristics				
Age ≥ 70 years [*n* (%)]	32 (68.09)	35 (47.95)	4.322	0.038
Male [*n* (%)]	31 (65.96)	36 (49.32)	2.876	0.090
BMI ≥ 25 kg/m^2^ [*n* (%)]	26 (55.32)	28 (38.36)	3.024	0.082
Disease-related factors				
Disease duration ≥ 5 years [*n* (%)]	22 (46.81)	18 (24.66)	5.876	0.015
NYHA class III-IV [*n* (%)]	28 (59.57)	20 (27.40)	11.274	0.001
Concomitant diabetes [*n* (%)]	28 (59.57)	27 (36.99)	5.393	0.020
Concomitant atrial fibrillation [*n* (%)]	24 (51.06)	17 (23.29)	8.929	0.003
Concomitant hypertension [*n* (%)]	38 (80.85)	51 (69.86)	1.611	0.204
Laboratory parameters				
LVEF < 40% [*n* (%)]	35 (74.47)	32 (43.84)	10.095	0.001
BNP ≥ 400 pg/mL [*n* (%)]	32 (68.09)	28 (38.36)	9.261	0.002
Creatinine ≥ 100 μmol/L [*n* (%)]	22 (46.81)	18 (24.66)	5.876	0.015
Serum sodium < 135 mmol/L [*n* (%)]	8 (17.02)	3 (4.11)	4.926	0.026
Hemoglobin < 120 g/L [*n* (%)]	18 (38.30)	12 (16.44)	6.732	0.009
Therapeutic factors				
Routine health education [*n* (%)]	31 (65.96)	31 (42.47)	5.751	0.017
No use of β-blockers [*n* (%)]	15 (31.91)	10 (13.70)	5.344	0.021
No use of ACEI/ARB [*n* (%)]	9 (19.15)	8 (10.96)	1.502	0.220

**Table 5:** Multivariable logistic regression analysis of factors associated with composite endpoint events.

Associated factors	B	S.E	Wald	P	OR	95% CI
NYHA class III-IV	1.326	0.425	9.723	0.002	3.765	1.638–8.654
Concomitant atrial fibrillation	0.892	0.398	5.024	0.025	2.440	1.119–5.318
LVEF < 40%	1.158	0.418	7.675	0.006	3.184	1.403–7.227
BNP ≥ 400pg/mL	0.784	0.402	3.806	0.051	2.190	0.996–4.814
Routine health education	0.958	0.386	6.158	0.013	2.607	1.223–5.558
Concomitant diabetes	0.726	0.378	3.685	0.055	2.067	0.986–4.334
Disease duration ≥ 5 years	0.645	0.398	2.622	0.105	1.906	0.874–4.158
Hemoglobin < 120 g/L	0.812	0.425	3.653	0.056	2.252	0.979–5.176

## 4 Discussion

This retrospective cohort study investigated the association between a stepwise health education model based on EBP and clinical outcomes in patients with CHF. The findings showed that this educational strategy was associated with better self-care behaviors, cardiac function, and selected clinical outcomes, providing a basis for future prospective studies and optimization of health education strategies. In this study, only the total score of EHFScB-9 at 6 months was designated as the primary outcome. All other outcomes and individual scale items were considered exploratory analyses and were performed without adjustment for multiple comparisons; therefore, isolated nominally significant findings should be interpreted with caution.

### 4.1 Association between stepwise health education based on EBP and cardiac function indicators

The findings of this study indicated that after 6 months of intervention, patients in the IG demonstrated significantly higher LVEF and 6MWD compared with those in the CG, while exhibiting significantly lower BNP levels. It suggests that this educational strategy is associated with better cardiac function indicators. Previous studies (17) have indicated that LVEF is an important indicator for evaluating left ventricular systolic function, and its improvement reflects the recovery of cardiac pumping function. As a biomarker of heart failure, the decreased level of BNP indicates reduced cardiac load and improved cardiac function. 6MWD is a simple and reliable indicator for evaluating exercise tolerance, and its increase reflects the improvement of the patient's overall functional status. Based on the research findings, the mechanisms underlying cardiac function improvement in the IG may involve multiple aspects: (1) Systematic health education enhanced medication adherence among patients, ensuring standardized use of guideline-recommended drugs, particularly the increased use rate of *β*-blocker, which plays a crucial role in improving ventricular remodeling and enhancing ejection fraction (18). (2) Lifestyle management such as salt and fluid restriction reduces the preload and afterload on the heart and facilitates cardiac function recovery. (3) Symptom monitoring and early intervention prevented acute exacerbations of heart failure and halted further deterioration of cardiac function. (4) Moderate exercise training improves skeletal muscle function and peripheral circulation, thereby indirectly reducing cardiac burden (19).

### 4.2 Association between stepwise health education based on EBP and clinical outcome optimization

This study found that the IG demonstrated significantly lower readmission rates at 30 days, 90 days, and 6 months compared to the CG, with a nearly 50% reduction in the incidence of the composite endpoint events. This finding is of great clinical significance. Previous clinical practice (20) has shown that the high readmission rate of CHF patients has long been a challenge in clinical management, which not only increases patient suffering and economic burden, but also consumes substantial healthcare resources. The reasons for the reduced readmission rate in the IG in this study mainly include the following: (1) The stepwise education model ensured systematic knowledge impartation and progressive deepening, enhancing patients’ disease awareness and self-care behaviors. (2) Symptom diaries and weight monitoring helped patients promptly identify early warning signs such as fluid retention, enabling early intervention. (3) Continuous reinforcement education after discharge sustained patients’ healthy behaviors, preventing knowledge loss and behavioral regression. (4) The establishment of a family support system provided continuous supervision and support, improving patient adherence (21, 22).

### 4.3 Association between stepwise health education based on EBP and biochemical indicators

The results of this study also revealed that at 6 months of intervention, serum sodium levels in the IG were significantly higher than those in the CG. A review of the literature (23) indicates that hyponatremia is a common electrolyte disturbance in CHF patients and is often closely related to poor patient prognosis. The improvement in serum sodium levels observed in the IG in this study may be attributed to the following factors: (1) Appropriate fluid restriction guidance prevented dilutional hyponatremia caused by excessive fluid restriction. (2) Standardized diuretic use reduced the incidence of electrolyte disturbances. (3) Nutritional guidance improved the overall nutritional status of patients. (4) Symptom control reduced neurohumoral activation, thereby improving water and sodium metabolism (24, 25). However, as these findings were derived from multiple unadjusted comparisons within the same outcome domain, the possibility of chance-positive results cannot be excluded. Therefore, they should be regarded as hypothesis-generating findings.

### 4.4 Exploratory findings on healthcare resource utilization during hospitalization

During hospitalization, the IG had a shorter average length of stay and lower total hospitalization costs than the CG; however, ICU admission rates and complication rates did not differ significantly between the groups. Because the health education was initiated during this hospitalization, length of stay, hospitalization costs, and early ICU admission were simultaneously influenced by baseline disease severity, treatment intensity, and healthcare resource utilization. Therefore, the observed between-group differences cannot be directly attributed to the educational strategy. These indicators were considered exploratory outcomes in this study, and their health economic implications require further validation in prospective studies with prespecified outcomes and adjustment for disease severity.

### 4.5 Significance of drug use optimization

This study showed that the use rate of *β*-blockers in the IG was significantly higher than that in the CG at 6 months of intervention. The finding has important guiding significance for clinical practice. In clinical practice, *β*-blockers are the cornerstone medications for CHF treatment and have been proven by multiple studies (26, 27) to improve prognosis and reduce mortality in CHF patients. However, due to concerns about side effects or insufficient awareness, the actual clinical use rate of *β*-blockers among some patients is often suboptimal. The stepwise health education based on EBP implemented in this study enabled patients to understand the importance of medications and their proper usage through systematic health education, thereby improving medication adherence. This plays a crucial role in enhancing long-term prognosis.

### 4.6 Stepwise health education based on EBP and self-care behaviors

The results of this study showed that at 6 months after intervention, the IG had significantly lower EHFScB-9 total scores and individual item scores than the CG. Given that lower EHFScB-9 scores indicate better self-care behaviors, these findings suggest that patients in the IG demonstrated better self-care practices related to symptom monitoring, medication adherence, and lifestyle management. Stepwise education may promote more stable self-care behaviors by progressively explaining the relationship between weight changes and fluid retention, providing simple recording methods, and reinforcing appropriate behaviors through feedback (14, 21). However, given the retrospective design, the influence of unmeasured factors, such as patient motivation, family support, and health literacy, cannot be excluded.

### 4.7 Clinical significance of analysis of factors associated with composite endpoint events

Multivariable analysis revealed that NYHA class III–IV, concomitant atrial fibrillation, LVEF <40%, and non-receipt of stepwise health education based on EBP were independently associated with composite endpoint events. The first three factors suggest the need for enhanced monitoring and disease management among high-risk patients. The association involving the educational strategy variable indicates that routine health education may require further improvement; however, health education itself should not be interpreted as a biological risk factor. This association may also be influenced by insufficiently measured factors, such as health literacy, family support, and accessibility of care, and requires further validation in prospective studies.

### 4.8 Research innovation, limitations, and future prospects

This study integrated EBP principles with a stepwise health education model and evaluated its association with self-care behaviors, cardiac function, and clinical outcomes in patients with CHF. However, this study also has certain limitations. First, the retrospective design may have introduced information bias, selection bias, and residual confounding, preventing the establishment of causal relationships. Second, the single-center setting and relatively small sample size may limit the generalizability of the findings. Third, social determinants such as health literacy, economic and care accessibility, and family support were not systematically collected. In addition, the educational program was not prospectively documented according to a specific named EBP framework before study initiation, nor was it independently validated as a standardized instrument; therefore, documentation of intervention dose and implementation consistency was limited. Index hospitalization outcomes overlapped temporally with the initiation of the educational intervention and could only be interpreted as exploratory findings. The follow-up duration was limited to 6 months. Furthermore, multiple exploratory analyses were conducted without adjustment for multiple comparisons, raising the possibility of cumulative type I error. Finally, the current continuous outcome analyses did not fully account for within-subject repeated measurements, and the primary outcome requires confirmation using baseline-adjusted ANCOVA based on the original individual-level data. Future studies should adopt clearly defined EBP frameworks, employ multicenter prospective designs, establish prespecified measures of educational fidelity, incorporate social determinants and patient-reported outcomes, and extend follow-up duration.

In summary, in this single-center retrospective cohort study, a stepwise health education approach based on EBP was associated with better self-care behaviors, cardiac function, and lower rates of readmission and composite endpoint events among patients with CHF. The approach integrates essential safety information with individualized delivery according to patients’ abilities, preferences, and clinical contexts, suggesting potential value for clinical process improvement. However, its causal effects and generalizability remain to be established through multicenter prospective studies.

## Data Availability

The original contributions presented in the study are included in the article/Supplementary Material, further inquiries can be directed to the corresponding author.
